# Faster gait speeds reduce alpha and beta EEG spectral power from human sensorimotor cortex

**DOI:** 10.1109/TBME.2019.2921766

**Published:** 2019-06-13

**Authors:** Andrew D. Nordin, W. David Hairston, Daniel P. Ferris

**Affiliations:** Department of Biomedical Engineering at the University of Florida, Gainesville, FL, USA; Human Research and Engineering Directorate at the U.S. Army Research Laboratory, Aberdeen, MD, USA; Department of Biomedical Engineering at the University of Florida, Gainesville, FL, USA

**Keywords:** electroencephalography, electromyography, independent component analysis, legged locomotion

## Abstract

**Objective:**

Our aim was to determine if walking speed affected human sensorimotor electrocortical dynamics using mobile high-density electroencephalography (EEG).

**Methods:**

To overcome limitations associated with motion and muscle artifact contamination in EEG recordings, we compared solutions for artifact removal using novel dual layer EEG electrodes and alternative signal processing methods. Dual layer EEG simultaneously recorded human electrocortical signals and isolated motion artifacts using pairs of mechanically coupled and electrically independent electrodes. For electrical muscle activity removal, we incorporated electromyographic (EMG) recordings from the neck into our mobile EEG data processing pipeline. We compared artifact removal methods during treadmill walking at four speeds (0.5, 1.0, 1.5, and 2.0 m/s).

**Results:**

Left and right sensorimotor alpha and beta spectral power increased in contralateral limb single support and push off, and decreased during contralateral limb swing at each speed. At faster walking speeds, sensorimotor spectral power fluctuations were less pronounced across the gait cycle with reduced alpha and beta power (*p*<0.05) compared to slower speeds. Isolated noise recordings and neck EMG spectral power fluctuations matched gait events and showed broadband spectral power increases at faster speeds.

**Conclusion and significance:**

Dual layer EEG enabled us to isolate changes in human sensorimotor electrocortical dynamics across walking speeds. A comparison of signal processing approaches revealed similar intrastride cortical fluctuations when applying common (e.g. Artifact Subspace Reconstruction) and novel artifact rejection methods. Dual layer EEG, however, allowed us to document and rule out residual artifacts, which exposed sensorimotor spectral power changes across gait speeds.

## I. Introduction

ELECTROENCEPHALOGRAPHY (EEG) is a non-invasive, lightweight and portable neuroimaging method with fast time scale for studying human electrocortical dynamics. Unfortunately, speed related changes in human electrical brain activity have been challenging to study because of motion artifact contamination at fast gait speeds [1]–[3]. Neural pathways between cortical motor planning centers and spinal cord circuits have also been a source of contention [4], with gait speed changes attributed to subcortical structures that can require limited cortical input [5].

Gait speed adjustments have been studied across species using invasive recordings from cortical and subcortical structures. Locomotion speed control has been traced to the mesencephalic locomotor region of the midbrain, which responds to electrical stimulation by initiating gait and proportionally increasing gait speed [5]–[7]. Recently, however, slow and fast gait speed mechanisms have been dissociated in mice by Caggiano et al. [8] and Josset et al. [9]. Separate neuronal populations were identified within pedunculopontine nucleus for controlling slow speeds and cuneiform nucleus for fast speeds. Separate gait speed control mechanisms therefore appear to project from these structures through the brainstem via lateral paragigantocellular nucleus and ultimately to the spinal cord [5], [8], [10].

Although gait speed is modulated by the mesencephalic locomotor region, presynaptic inputs to pedunculopontine nucleus are received from basal ganglia and medulla, and cuneiform nucleus receives input from the periaqueductal grey and inferior colliculus [5]. Inhibitory mesencephalic inputs are also received from central amygdala, superior colliculus and dorsal raphe [5], [11]. Motor cortex, however, has input into basal ganglia, which appears to relay into pedunculopontine nucleus during slow locomotor control [5]. To understand the role of motor cortex during gait, its activity has therefore been studied across the stride and during locomotor adjustments.

Neuronal activity in motor cortex during animal locomotion has revealed fluctuations across the gait cycle. Studies in cats by Drew et al. [12], [13] and Beloozerova et al. [14], [15] have shown increased motor cortex activity during forelimb swing, which further increased during precision stepping. Studies in rats, however, showed hindlimb locomotion can be largely controlled subcortically [16], [17], though gait timing and limb kinematics can be decoded from cortical activity [18]–[20]. Recently, DiGiovanna and colleagues [21] showed neuronal firing in rat motor cortex more closely resembles activation patterns in cats than previously thought. Specifically, motor cortex firing preceded gait initiation, fluctuated with hindlimb trajectories and muscle activities, and decreased in more automated behaviors, such as treadmill stepping [21]. Studies in non-human primates have shown similar instrastride fluctuations that are highly structured and reproducible across gait speeds [22]–[24]. Within specific gait phases, however, locomotion speed has shown little effect on motor cortex firing rates in cats [25], [26] and rats [21], and non-human primates have shown mixed trends [22]–[24]. Although human sensorimotor alpha (8–13 Hz) and beta band (13–30 Hz) cortical oscillations have shown reduced spectral power during upper and lower limb motor preparation and execution [27]–[30], and at the instant of gait speed adjustments in slow treadmill walking [31], spectral power changes at faster walking speeds remain unclear.

Many human EEG studies have now reported electrocortical fluctuations across the gait cycle [32]–[39]. Gwin and colleagues [32] first identified gait related spectral fluctuations in left and right sensorimotor cortices, along with anterior cingulate and posterior parietal cortices. The authors [32] showed alpha and beta spectral power increases during double support and decreases during limb swing, but the appearance of broadband (3–150 Hz) spectral power fluctuations in each cortical cluster could relate to motion or muscle artifacts [1], [2], [32]. To limit EEG artifacts during locomotion, human brain dynamics have therefore largely been studied in slow walking and gait-like tasks [32]–[42]. Although scalp EEG recordings are prone to artifacts arising from electrode and cable motions, as well as confounding electrophysiological signals (e.g. eye and muscle) and environmental electrical noise, recent hardware and signal processing advances have expanded possibilities for studying electrical brain signals in dynamic tasks [43], [44]. Dual layer EEG hardware that simultaneously records electrocortical signals along with isolated noise from secondary sensors can enhance signal processing efforts for noise removal and help rule out the influence of noise artifacts in EEG recordings during locomotion [43], [44]. Capabilities and best practices for dual layer EEG processing, however, have yet to be established for human EEG recordings. Comparisons among common and novel signal processing approaches using dual layer EEG hardware are therefore needed for removing electrical, mechanical, and biological artifacts from mobile EEG.

Our aim was to study human electrocortical dynamics across a range of gait speeds using mobile EEG. Because motion and muscle artifacts have imposed barriers to the collection and interpretation of human scalp EEG at fast gait speeds, we evaluated traditional and novel processing approaches for motion and muscle artifact removal using dual layer EEG. We hypothesized that alpha and beta EEG spectral power would increase during double support and decrease during limb swing, independent of gait speed, and that dual layer EEG would allow motion and muscle artifacts to be quantified and removed through signal processing. Dual layer EEG artifact removal may then uncover gait speed-related changes in human EEG spectral power.

## II. Methods and materials

Prior to participation, nine healthy subjects (6M, 3F, mean age 27 ± 4 years) provided institutionally approved informed consent. Institutional Review Boards at the University of Michigan and University of Florida approved the study. To begin each collection, subjects were fit with an appropriately sized 128-channel EEG cap and the location of each scalp electrode was measured with a Zebris digitizing system. After participation, each subject received an anatomical magnetic resonance image used during EEG source localization.

### A. Dual layer EEG hardware

**Figure 1.**
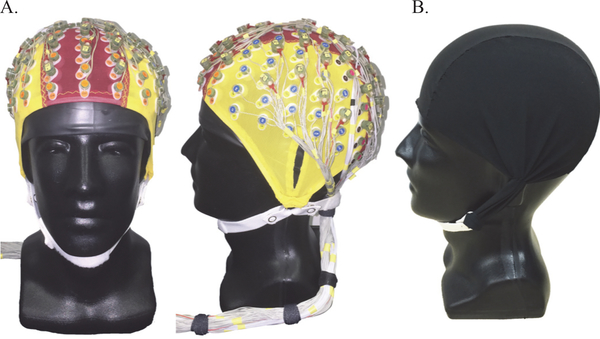
Dual layer EEG displayed on a mannequin head (A.). 128-channel scalp interfacing EEG electrodes and 40 mechanically coupled and inverted noise-only electrodes bundled into a dual layer EEG array. Noise-only sensors were referenced to an overlaid custom conductive fabric cap (B.).

Our dual layer EEG array consisted of 128-scalp interfacing EEG electrodes, with 40 mechanically coupled and inverted noise-only electrodes that were electrically isolated from the primary scalp EEG sensors [43], [44] (Fig. 1). The 128-scalp EEG electrodes were pin type BioSemi ActiveTwo sensors that fit into a standard 128-channel EEG cap after applying conductive gel into each electrode well. The 40-noise electrodes were flat type BioSemi ActiveTwo sensors that were paired with scalp sensors evenly distributed across the EEG cap. Wires from each EEG-noise pair were bundled with each other electrode wire, forming a single cable bundle exiting the rear of the EEG cap (Fig. 1A). To serve as an electrically isolated artificial skin circuit for the noise electrodes, a custom conductive fabric cap (Eeonyx, Fig. 1B) was fit over the inverted noise sensors, which approximately matched the resistivity of human skin [44]. Conductive gel was inserted between the conductive fabric and the inverted recording electrode to complete the artificial skin circuit. Eight flat type BioSemi ActiveTwo sensors were also placed on the left and right sternocleidomastoid and trapezius muscles (2 electrodes per muscle), capturing EMG activity from the neck. In total, the 128-scalp EEG and 8-neck EMG electrodes were collected from a single BioSemi collection box and the 40-noise electrodes were collected from a separate BioSemi collection box. The two systems were daisy-chained, which stored the EEG, EMG, and noise data in a single synchronized data file sampled at 512 Hz. During testing, the BioSemi collection boxes were placed above the subject on a bodyweight support apparatus.

### B. Experimental protocol

Subjects completed testing in randomized walking speed conditions (0.5, 1.0, 1.5, 2.0 m/s) on a Bertec force-instrumented treadmill used to detect heel strike and toe off events for each limb. During testing, additional experimental conditions were completed, but we focus this analysis on continuous walking conditions at different speeds. Each speed conditions were completed, but we focus this analysis on continuous walking conditions at different speeds. Each speed condition lasted 3-minutes with rest periods between. Subjects were instructed to walk normally while restricting unnecessary eye blinks, head motions, or jaw clenching. Because our fastest locomotion condition was near the preferred human walk to run transition speed [45], we asked subjects to remain walking in each condition. Prior to gait speed conditions, a standing baseline trial was also recorded.

### C. EEG processing

**Figure 2.**
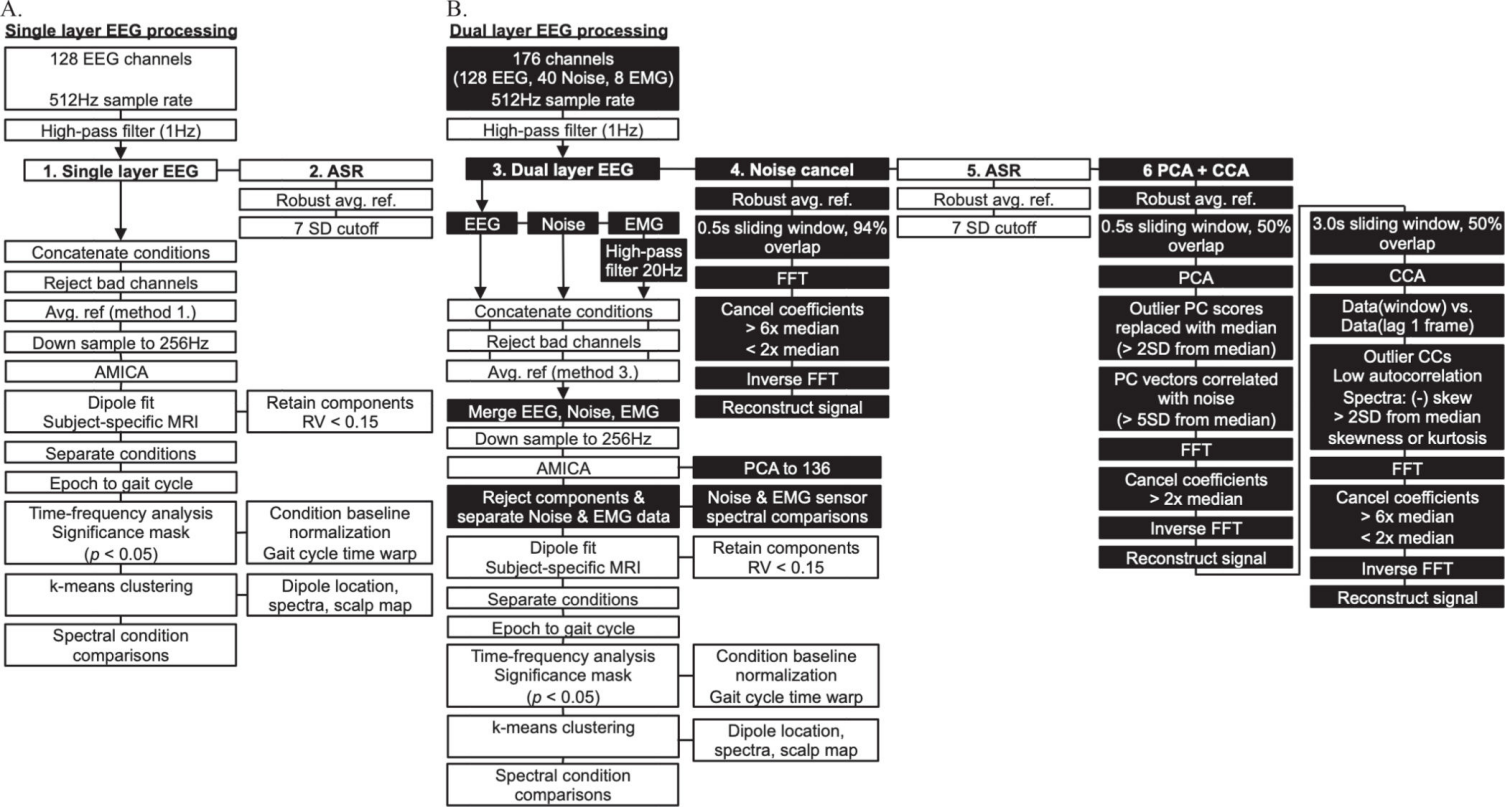
Single (A.) and dual layer EEG (B.) processing pipelines. Common EEG processing steps (white boxes) and dual layer specific EEG processing steps (black boxes). Single-layer EEG approaches relied on 128-scalp EEG electrodes without additional preprocessing (1: Single layer EEG) and after Artifact Subspace Reconstruction (2: ASR). Dual-layer EEG approaches relied on 128-scalp EEG electrodes, 40 isolated noise-only electrodes, and 8 neck EMG electrodes merged into an adaptive mixture independent component analysis (AMICA) after contrasting preprocessing steps. Dual-layer EEG processing was completed without additional preprocessing (3: Dual layer EEG), after frequency domain noise cancellation (4: Noise cancel), after ASR (5), and after applying frequency domain noise cancellation to artifact components from principal component analysis and canonical component analysis (6: PCA+CCA).

Figure 2 illustrates our EEG processing pipeline used to isolate and fit electrocortical sources to each subject’s brain, perform time-frequency and spectral analyses, and statistical testing in EEGLab [46]. We evaluated multiple EEG processing procedures that included two single layer EEG methods (Fig. 2A) and four dual layer EEG methods (Fig. 2B) using EEGLab functions and custom MATLAB scripts. To distinguish traditional single layer EEG processing from our dual layer EEG approach, we highlighted novel processing steps in black boxes (Fig. 2B). Single and dual layer EEG processing differed in the number of channels analyzed, data preprocessing, independent component analysis input, and independent component rejection. Otherwise, common EEG processing steps are shown in white boxes (Fig. 2).

Common EEG processing steps involved high-pass filtering channel data (1Hz) followed by preprocessing. Next, data from each speed condition were concatenated and outlier channels were rejected using statistical criteria (kurtosis and standard deviation) [32]. In methods without preprocessing, EEG data were common average referenced after channel rejection (methods 1 & 3, Fig. 2). A robust average reference was applied before each other preprocessing method by excluding outlier channels from the average. Prior to performing adaptive mixture independent component analysis (AMICA) [47], we down sampled data to 256 Hz. We then modeled independent components as equivalent current dipoles using a three layer boundary element model and subject-specific anatomical magnetic resonance image warped to the Montreal Neurological Institute standard brain using DIPFIT and Fieldtrip function in EEGLab [48]. Dipoles with residual variance less than 0.15 were retained for further analysis. We extracted complete gait cycles from our EEG data using synchronized vertical ground reaction force gait events, delimited by right heel strike. Time-frequency analysis was performed using single trial spectrograms that were baseline normalized within each speed condition, and time-warped to create event related spectral perturbation (ERSP) plots across the gait cycle [32], [50]. Group analysis relied on k-means clustering using vectors jointly coding dipole locations, scalp maps, and spectral power similarities in EEGLab [40], [49]. Clusters containing multiple independent components per subject were first aggregated within subjects to avoid artificially inflating sample size during statistical testing [44]. We then averaged time-frequency data across subjects in each cluster. Next, we set non-significant spectral power changes to zero using bootstrap methods in EEGLab (α = 0.05). Cortical clusters with components from more than 50% of the subjects were further analyzed. Because we compared multiple EEG processing methods, we focused our analysis on consistent cortical clusters with spectral fluctuations across the gait cycle. Finally, we evaluated spectral power differences among speed conditions using non-parametric bootstrap-based ANOVA in EEGLab (α = 0.05).

### D. Single layer EEG processing

Single layer EEG processing (Fig. 2A) was completed without additional preprocessing (1: Single layer EEG) and after artifact subspace reconstruction (2: ASR) in EEGLab. ASR is a commonly used EEG preprocessing method that relies on 0.5 s sliding window principal component analysis to correct and reconstruct non-stationary high variance EEG data based on statistical criteria from clean EEG [51]. Here, we used a standing baseline for each subject during ASR and applied a 7 standard deviation cutoff based on pilot testing. The remainder of each single layer EEG processing approach was performed as outlined above and in Fig. 2A using data from the scalp interfacing dual layer EEG sensors and omitting data from the outer layer sensors (Fig. 1).

### E. Dual layer EEG processing

Dual layer EEG processing (Fig. 2B) was completed without additional preprocessing (3: Dual layer EEG), after frequency domain noise cancellation (4: Noise cancellation), after artifact subspace reconstruction (5: ASR), and after applying frequency domain noise cancellation to artifact components from principal component analysis and canonical component analysis (6: PCA+CCA). Each dual layer EEG preprocessing approach relied on methods adapted from Nordin et al. [44], using similar AMICA input and independent component rejection steps. In each case, dual layer EEG preprocessing output was merged with 40-channel dual layer noise data and 8-channel EMG data by stacking channels. We reduced dual layer EEG AMICA output to 136 dimensions using PCA. To reject dual layer EEG independent components, component spectra were compared to noise and EMG channels using a polynomial fit. Components with flat spectra (linear slope ≥ −0.06) or those matching noise or muscle (*R*^*2*^ ≥ 0.99) were rejected.

Frequency domain noise cancellation was applied using methods from Nordin et al. [43]. This method cancels artifacts from scalp-interfacing EEG electrodes using artifacts captured by dual layer noise electrodes. Because our array consisted of 128-EEG electrodes and 40-matched noised pairs, we used spherical interpolation in EEGLab to compute matched noise pairs for all 128-EEG electrodes. We then used the noise cancellation algorithm to separately perform Fast Fourier Transform (FFT) on the EEG and noise data in a 0.5 s sliding window with 94% overlap. Noise frequencies in the EEG signal were cancelled using cutoffs based on the median Fourier coefficients across frequencies, and the signal was reconstructed using inverse FFT [43]. We used separate upper (>6x median) and lower cutoffs (<2x median) for motion artifact and electrical noise cancellation, respectively. To account for magnitude differences between EEG and noise signals, we scaled noise FFT coefficients to the median EEG FFT coefficients and reconstructed an amplitude-matched noise signal, which we later used in dual layer AMICA. Our aim was to match noise and EEG artifact signal amplitudes, compensating for resistivity differences between the scalp and conductive fabric. This overall approach has outperformed direct time or frequency domain noise subtractions when applying algorithm parameters from pilot testing.

To remove motion and muscle artifacts from our dual layer EEG data, we combined preprocessing approaches. Because ASR relies on principal component analysis to reconstruct signal components that deviate from clean EEG data, we developed a process to clean large variance components based on comparisons to dual layer noise electrodes. To do so, we used a 0.5 s sliding window with 50% overlap to perform PCA on the EEG channel data. We replaced outlier PC scores (>2SD from the median) with the median and cleaned components highly correlated with 40-channel dual layer noise mean (>5SD from the median noise correlation) using frequency domain noise cancellation (cancel Fourier coefficients >2x noise median). The signal was then reconstructed from artifact cleaned principal components.

Next, because canonical component analysis has been used to remove EEG motion and muscle artifacts [52], [53], we used a 3.0 s sliding window with 50% overlap to perform CCA on the PCA preprocessed EEG data. CCA input relied on channel data with a 1-frame lag autocorrelation, which separates low frequency, high autocorrelation motion artifact components from high frequency, low autocorrelation electrical and muscle artifact components. Components with low autocorrelation (below the component-autocorrelation plot knee) or biased power spectra (negatively skewed: high frequency, or outlier skewness or kurtosis: >2SD from the median) were cleaned using frequency domain noise cancellation (cancel Fourier coefficients >6x noise median or <2x noise median). The signal was then reconstructed from artifact cleaned canonical components. We found that using PCA before CCA to cancel large artifacts improved the performance of CCA for muscle and electrical artifact cancellation, as well as residual motion artifacts. Overall parameter selection was the result of pilot testing.

### F. Dual layer EEG noise and EMG channel processing

After analyzing our EEG data using each single and dual layer method, we performed similar channel-based analyses on the 40 dual layer noise channels and 8 neck EMG channels. The purpose was to evaluate pure motion and muscle artifacts by examining equivalent spectral power fluctuations across the gait cycle and changes in spectral power among speed conditions. We compared motion and muscle artifact signal changes to our preprocessed and ICA-derived electrocortical sources. Channel data were analyzed after high pass filtering at 1 Hz, down sampling to 256 Hz, extracting gait cycle epochs, and performing time-frequency analysis. ERSP plots were normalized to baseline within each speed condition and masked for significance (α = 0.05). Spectral power differences among speed conditions were also assessed using non-parametric bootstrap-based ANOVA in EEGLab (α = 0.05).

## III. Results

**Figure 3.**
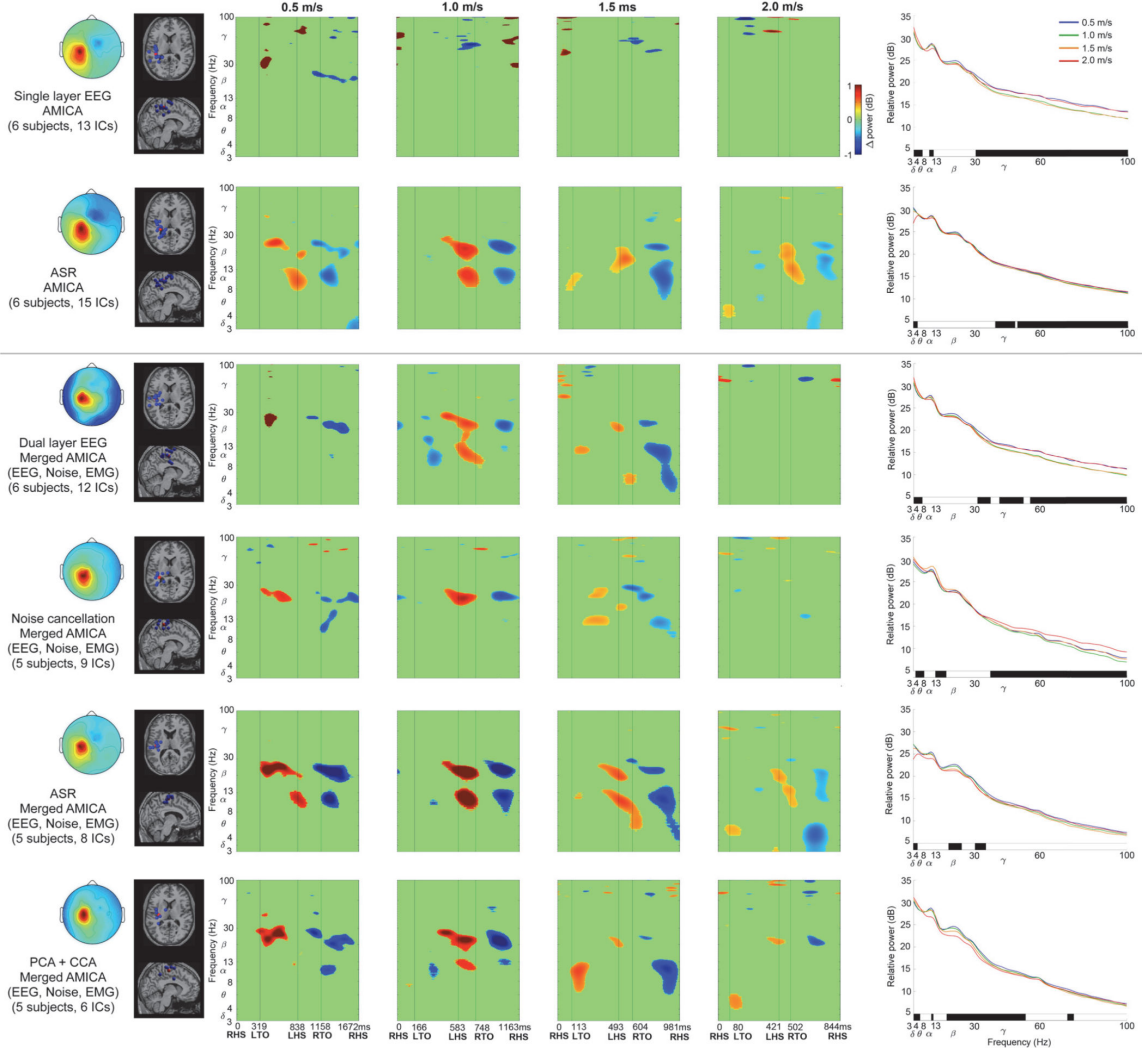
Left sensorimotor cortex data processing comparisons (separate processing procedure in each row). Two traditional single-layer EEG approaches were applied to the scalp interfacing dual layer EEG sensors (top two rows) and four dual-layer EEG approaches incorporated all sensor data (bottom four rows). Left to Right: Mean cluster topographic map, Dipole locations (Blue: subject dipoles, Red: cluster centroid), Event Related Spectral Perturbation plots at each walking speed (Left to right: slow to fast, significance masked: *p* < 0.05), Power spectral density at each walking speed (significant speed differences at each frequency identified below each plot in black, *p* < 0.05). ERSP plots without statistical significance masking are in Supplementary Fig. A.

**Figure 4.**
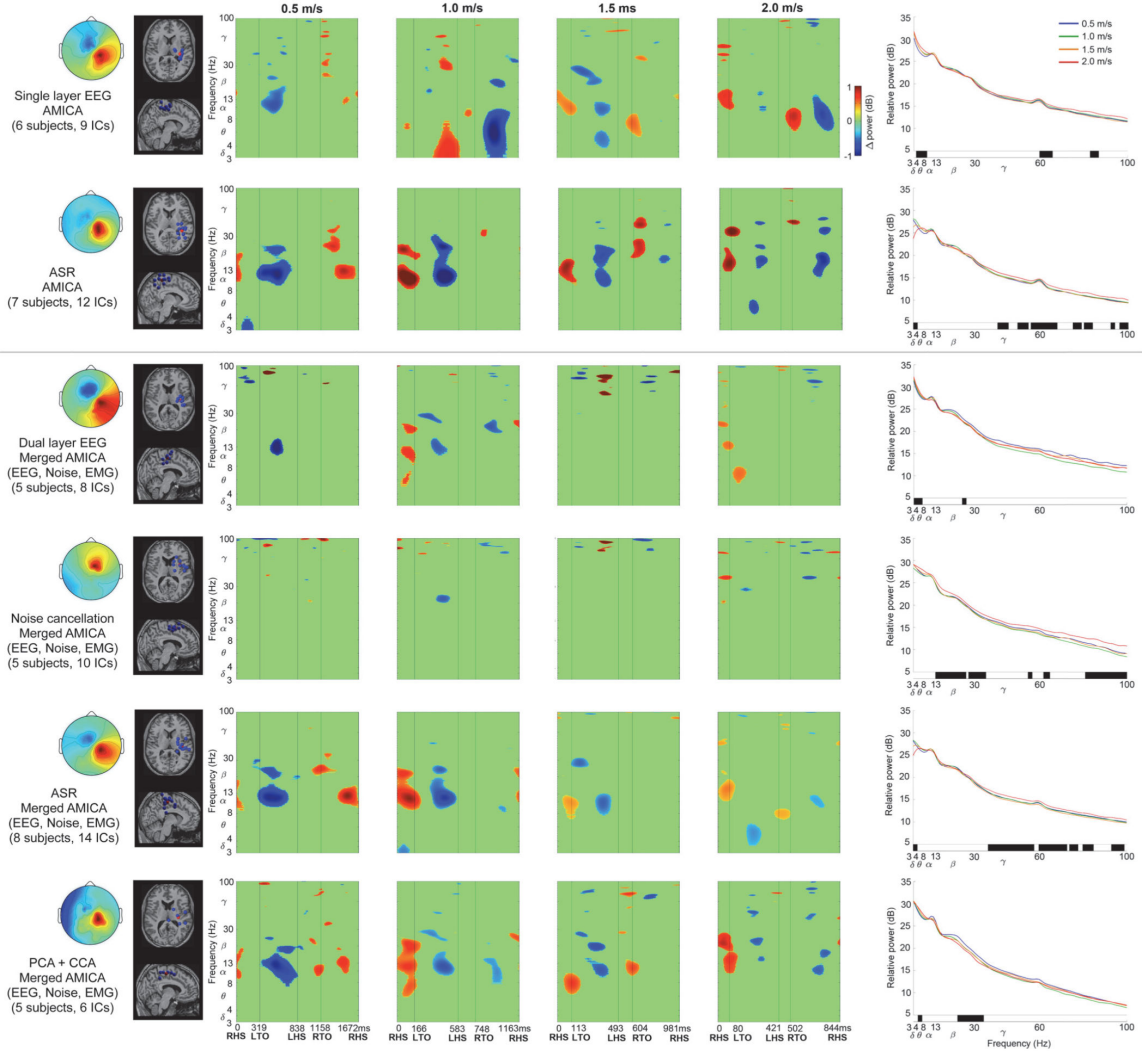
Right sensorimotor cortex data processing comparisons (separate processing procedure in each row). Two traditional single-layer EEG approaches were applied to the scalp interfacing dual layer EEG sensors (top two rows) and four dual-layer EEG approaches incorporated all sensor data (bottom four rows). Left to Right: Mean cluster topographic map, Dipole locations (Blue: subject dipoles, Red: cluster centroid), Event Related Spectral Perturbation plots at each walking speed (Left to right: slow to fast, significance masked: *p* < 0.05), Power spectral density at each walking speed (significant speed differences at each frequency identified below each plot in black, *p* < 0.05). ERSP plots without statistical significance masking are in Supplementary Fig. B.

Left and right sensorimotor cortices showed spectral power fluctuations across the gait cycle using contrasting EEG processing methods. Figure 3 shows increased left sensorimotor alpha and beta spectral power surrounding left heel strike, and decreased alpha and beta spectral power after right toe off. Left sensorimotor alpha and beta fluctuations therefore increased during right limb single support and push off in double support, but decreased during right limb swing. In contrast, Figure 4 predominantly shows asynchronous spectral power fluctuations in right sensorimotor cortex compared to left. Right sensorimotor alpha and beta power increased surrounding right heel strike, followed by decreased alpha and beta power after left toe off. Right sensorimotor alpha and beta power therefore decreased during left limb swing, but increased during left limb single support and push off in double support. At faster gait speeds, electrocortical fluctuations were less pronounced across the gait cycle, with limited amplitude, duration, and spectral bandwidth compared to slow walking (Figs. 3 & 4, ERSPs). Supplementary Figures A and B show spectral power fluctuations from Figures 3 and 4 without significance masking.

Discrepancies were apparent among EEG processing approaches when assessing spectral power fluctuations across the gait cycle. Similarities were evident in the scalp map, dipole locations and ERSP plots among single layer and dual layer EEG processing using ASR (Figs. 3 & 4, rows 2 & 5, respectively) and dual layer PCA+CCA (Figs. 3 & 4, row 6). Each of these methods showed lateralized asynchronous sensorimotor electrocortical fluctuations across the gait cycle. In contrast, single and dual layer EEG without additional preprocessing (Figs. 3 & 4, rows 1 & 3, respectively) and noise cancellation (Figs. 3 & 4, row 4) did not show consistent gait-related spectral power fluctuations, despite relatively similar scalp topography and dipole locations compared to each other processing method.

Spectral power changes across speed conditions further exposed differences among EEG processing methods (Figs. 3 & 4, right column). Left sensorimotor cortex mostly showed increased gamma power at faster gait speeds (*p* < 0.05) based on single layer EEG and ASR (Fig. 3, rows 1 & 2, respectively), as well as dual layer EEG without preprocessing and after noise cancellation (Fig. 2, rows 3 & 4, respectively). In contrast, dual layer ASR and PCA+CCA showed reduced left sensorimotor beta power at faster gait speeds (Fig. 3, rows 5 & 6, *p* < 0.05), and PCA+CCA also showed reduced alpha power at faster speeds (Fig. 3, row 6, *p* < 0.05). Similar to the left, right sensorimotor cortex showed greater gamma power at faster speeds (*p* < 0.05) based on single layer EEG and ASR (Fig. 4, rows 1 & 2, respectively), as well as noise cancellation and dual layer ASR (Fig. 4, rows 4 & 5, respectively, *p* < 0.05). In contrast, PCA+CCA, showed reduced beta power at faster gait speeds (Fig. 4, row 6, *p* < 0.05).

**Figure 5.**
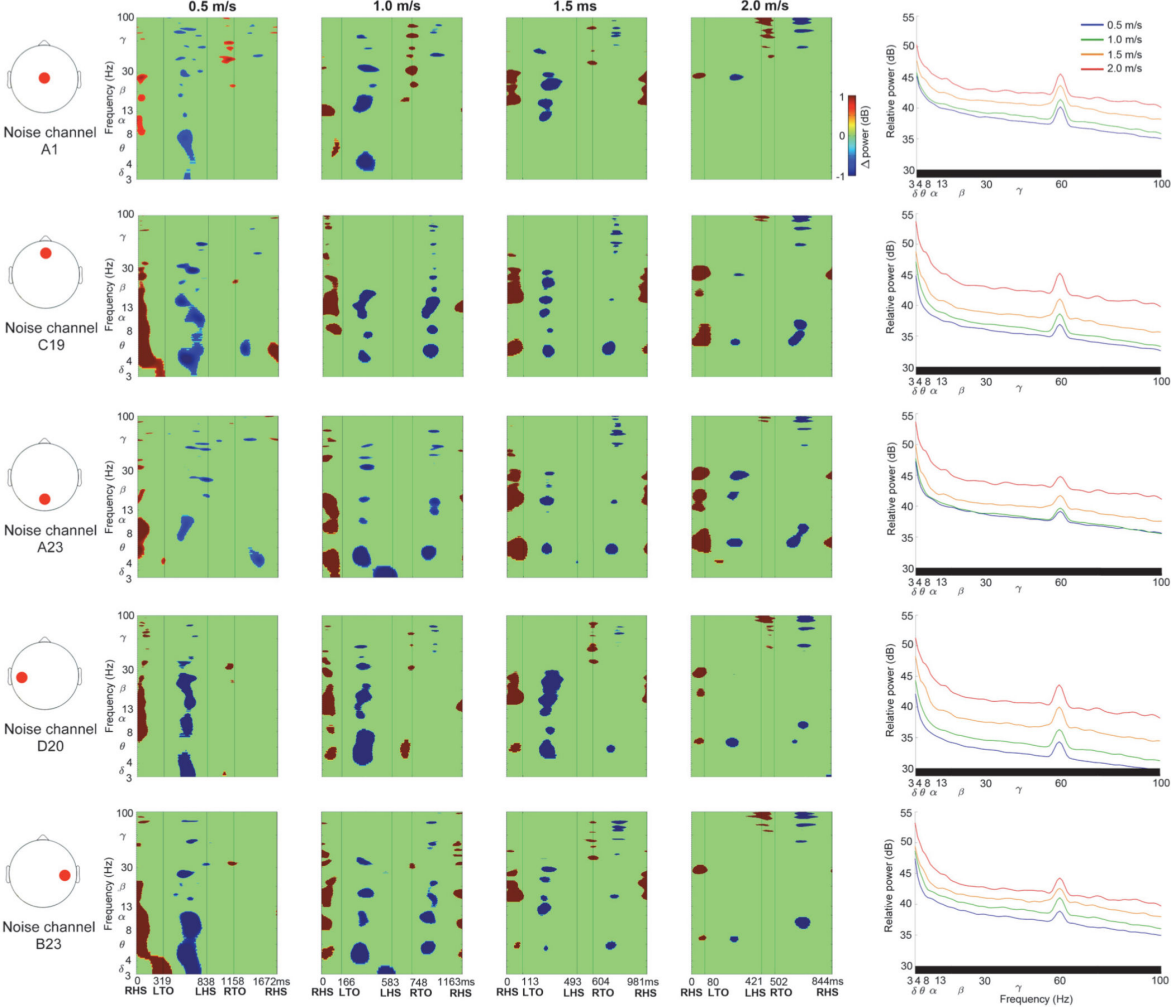
Exemplar dual electrode isolated noise channel data. Left to Right: Noise channel scalp location (red circle), Event Related Spectral Perturbation plots at each walking speed (Left to right: slow to fast, significance masked: *p* < 0.05), Power spectral density at each walking speed (significant speed differences at each frequency identified below each plot in black, *p* < 0.05). ERSP plots without statistical significance masking are in Supplementary Fig. C.

Dual electrode noise recordings captured spectral fluctuations due to motion artifacts across the gait cycle (Fig. 5). Noise fluctuations varied slightly across scalp locations, but consistently showed increased spectral power following heel strike, during double support, and reduced spectral power during swing, without lateralization. Artifact related broadband spectral power fluctuations are seen clearly in Supplementary Figure C, which shows Figure 5 ERSPs without significance masking. Spectral fluctuations tended to increase in amplitude at faster gait speeds (Fig. 5 & Supplementary Fig. C ERSPs), along with broadband spectral power increases (Fig. 5, right column, *p* < 0.05).

**Figure 6.**
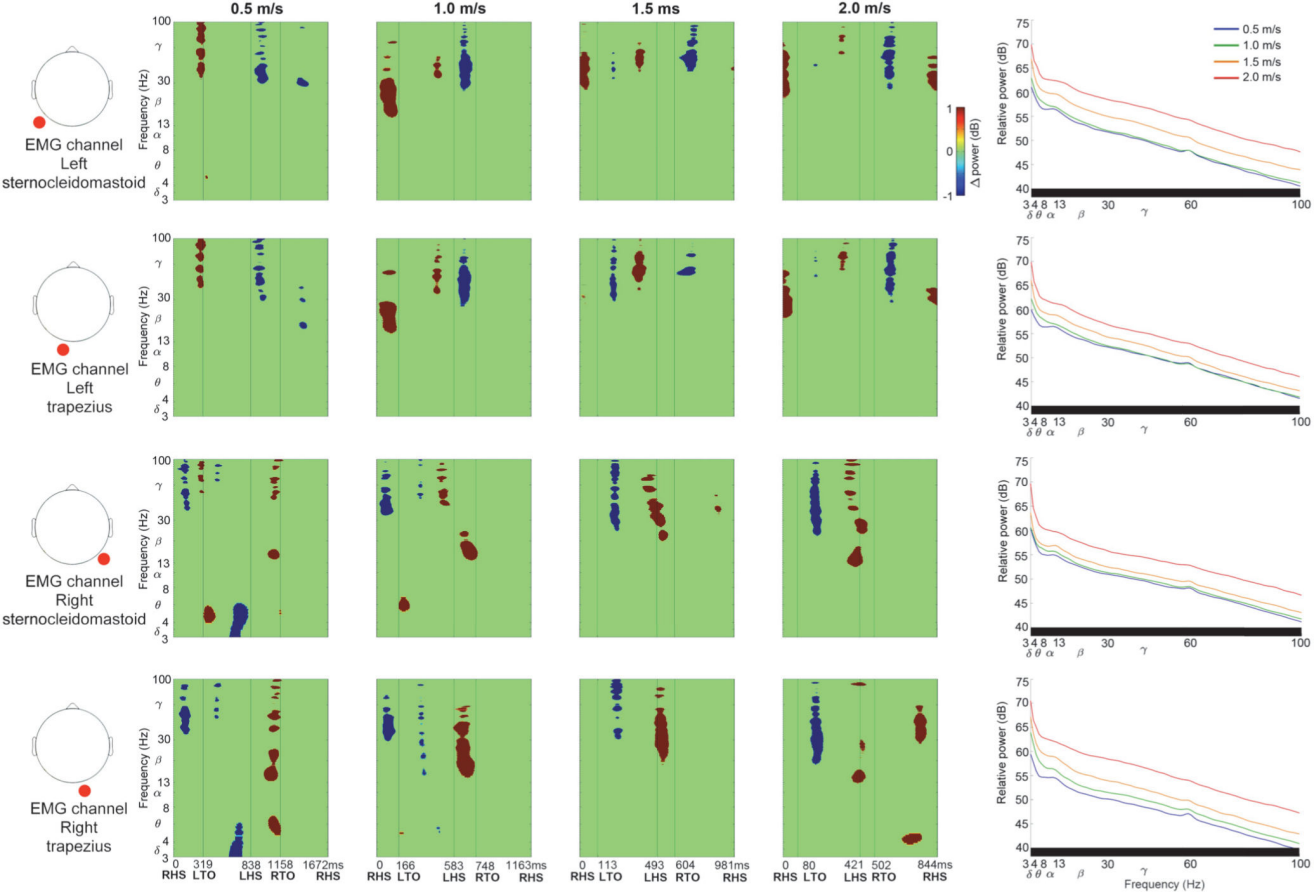
Exemplar neck EMG channel data (left and right sternocleidomastoid and trapezius muscles). Left to Right: EMG channel location (red circle), Event Related Spectral Perturbation plots at each walking speed (Left to right: slow to fast, significance masked: *p* < 0.05), Power spectral density at each walking speed (significant speed differences at each frequency identified below each plot in black, *p* < 0.05). ERSP plots without statistical significance masking are in Supplementary Fig. D.

Neck EMG recordings showed spectral power fluctuations across the gait cycle (Fig. 6). Each neck muscle recording contained lateralized high frequency gamma oscillations, and broadband spectral fluctuations were evident without significance masking (Supplementary Fig. D). Left and right sternocleidomastoid and trapezius muscles predominantly showed increased spectral power preceding heel strike and during double support with the contralateral limb leading, and prior to ipsilateral heel strike. Left and right neck muscles also showed decreased spectral power during double support with the ipsilateral limb leading, through swing. Lateralized neck EMG spectral power fluctuations did not match left and right sensorimotor fluctuations across the gait cycle, nor did they match dual electrode noise recordings. Neck EMG spectral fluctuations tended to increase at faster gait speeds (Fig. 6 & Supplementary Fig. D ERSPs, *p* < 0.05), along with broadband spectral power increases (Fig. 6, right column).

## IV. Discussion

We observed asynchronous spectral power fluctuations in left and right sensorimotor cortices across the gait cycle, with reduced duration and frequency bandwidth at faster gait speeds. Sensorimotor alpha and beta power increased during contralateral limb single support and push off, and decreased during contralateral limb swing. Mean spectral power across the gait cycle showed reduced left and right sensorimotor beta power, and reduced right sensorimotor alpha power, at faster gait speeds, after removing muscle artifacts. Gamma power increased in left and right sensorimotor cortices at faster gait speeds without removing EEG muscle artifacts, but did not show gait speed differences after EMG artifact removal.

By simultaneously collecting isolated noise recordings from our dual layer EEG electrodes, we were able to characterize spectral power fluctuations due to motion artifacts across the gait cycle at a range of speeds (Fig. 5). Motion artifact related spectral power fluctuations increased with gait speed and overlapped with left and right sensorimotor electrocortical fluctuations in Figures 3 and 4, which illustrates challenges involved in isolating brain signals from scalp EEG during locomotion. After preprocessing with ASR or PCA+CCA, however, we were able to identify robust lateralized sensorimotor cortical activity across a range of gait speeds that is distinct from isolated EEG motion artifacts and EMG recordings. Dual layer EEG therefore allowed us to rule out residual artifacts from our electrocortical sources.

Our observed changes in sensorimotor dynamics with gait speed are largely in agreement with invasive recordings during animal locomotion. Our human electrocortical spectral power fluctuations occurred within specific phases of the gait cycle that were maintained across locomotion speeds These data reflect similar neuronal firing rate patterns throughout the gait cycle in cats [25], [26], rats [21] and non-human primates [22]–[24], with neuronal spike rates that varied across the stride. Increased firing rates within the motor cortex have also been reported in transitions between single and double support [21], [22] and during limb swing [12], [13], [15], [24]. Our EEG data had spectral power increases in sensorimotor cortices during contralateral limb single support and push off, and decreases in swing. Compared to slower speeds, however, faster walking speeds had reduced spectral power fluctuation durations and frequency bandwidth in the gait cycle. Reduced overall sensorimotor alpha and beta power at faster gait speeds suggests greater cortical involvement compared to slow walking [54]. One explanation is that sensorimotor cortices are processing increased sensory feedback throughout the gait cycle at faster speeds. If sensorimotor cortex is more attuned to sensory feedback, it could be better primed for performing unexpected gait adjustments at fast speeds, such as stepping over obstacles [44]. Although animal studies have shown increased, decreased, and unchanging neuronal firing rates in sensorimotor cortex with changes in locomotion speed [21]–[26], comparisons between neuronal firing rates and EEG spectral power are indirect. Despite substantial evidence that locomotor speed is largely controlled subcortically [5]–[11], sensory integration involves many cortical structures [13]. Reduced alpha and beta EEG spectral power from human primary motor and parietal cortices at the instant of slow gait speed transitions (~0.3–0.6 m/s) have therefore exposed cortical contributions to gait speed adjustments [31].

Slow gait-like stepping tasks have previously shown sensorimotor electrocortical fluctuations measured with EEG. During slow robot assisted walking (~0.5–0.6 m/s), Wagner and colleagues [33] and later, Seeber et al. [34], [35], showed low gamma fluctuations (~24–40 Hz) in central sensorimotor areas, without lateralization, though task differences might present contrasting brain dynamics compared to unassisted gait. Bradford and colleagues [36] subsequently showed lateralized asynchronous spectral fluctuations in left and right sensorimotor cortices during level and incline walking at 0.75m/s, and Oliveira et al. [37] isolated similar activities in somatosensory cortices during walking at 1.0 m/s with eyes open and closed. In each case, alpha and beta power increased in double support during contralateral limb push off, and decreased during swing, in agreement with our results.

Bulea and colleagues [38] also studied slow (0.8–0.9 m/s) and fast treadmill walking (1.4–1.5 m/s), in active and passive speed control conditions, with some evidence of lateralized spectral fluctuations in left and right motor cortices in slow walking. The authors [38], however, applied ASR to their EEG data using an aggressive three standard deviation cutoff that can attenuate or remove brain signals along with artifacts. In separate studies, Luu et al. also applied ASR with a three standard deviation cutoff, but did not report lateralized sensorimotor activity during level walking, and ramp and stair ascent [41], nor while controlling an avatar during treadmill walking [42]. Recently, however, Artoni and colleagues [39] reported spectral power fluctuations across the gait cycle in cortical motor regions after applying ASR with 20 standard deviation cutoff. Similar to our results and previous studies, the authors showed spectral power decreases during limb swing and increases during double support, and the authors were able to identify unidirectional connectivity to lower limb muscles during limb swing, indicative of motor drive [39].

Although we report lateralized activity in sensorimotor cortices, gait related spectral fluctuations have been reported in several cortical areas in previous mobile EEG studies, including occipital lobe, supplementary motor area, anterior cingulate, posterior parietal, prefrontal, and premotor cortices [32], [36]–[39], [41], [42]. We therefore cannot rule out gait speed changes in other cortical structures, but restricted our analysis to clusters that appeared in multiple preprocessing methods, and with prominent spectral fluctuations across the gait cycle. The inclusion of additional tasks, contrasting EEG processing, electrode configurations, or residual motion and muscle artifacts, could also lead to the appearance of intrastride spectral fluctuations in other brain areas. Additional studies that apply preprocessing steps to remove artifacts while preserving electrocortical activity are therefore needed.

In addition to our reported spectral power fluctuation patterns across the gait cycle, mean spectral power revealed important electrocortical changes among gait speeds. Reduced sensorimotor alpha and beta power was observed at faster gait speeds, often coinciding with increased gamma power (Figs. 3 & 4, right column). Although multiple processing methods showed these trends, dual layer EEG preprocessing with PCA and CCA appeared to limit artifact related variability that masked statistical differences in alpha and beta bands, and broad gamma band increases that were similar to motion artifact and EMG recordings (Figs. 5 & 6, right column). Decreased sensorimotor alpha and beta spectral power are expected electrocortical responses during motor preparation and execution [27]–[30], but increased gamma power has also been reported in isolated upper and lower limb movements using electrocorticography, magnetoencephalography, and EEG [27], [55]–[57]. Observed spectral power differences among gait speeds could therefore indicate signal over cleaning when applying dual layer PCA and CCA for motion and muscle artifact removal [58]. Movement related gamma band activity, however, tends to be localized with short duration around movement onset and offset, which is difficult to record using scalp EEG because of its comparatively low signal to noise ratio and spectral overlap with muscle activity [55]–[57]. Nevertheless, increased primary motor cortex gamma power has been reported by McCrimmon et al. using electrocorticography during treadmill walking [59], and this activity showed intrastride fluctuations. The authors reported more gamma bursts across the gait cycle at faster walking speed, but this finding was limited to one of two epileptic patients in the study [59]. Intrastride fluctuations were also shown in alpha and beta bands for one subject [59], but the authors acknowledge the possibility of alpha band motion artifact contamination in their electrcortographic data. Because the scalp, skull, and dura mater can also blur and low pass filter electrocortical signals [57], [60], [61], representations of gait related electrocortigraphic gamma activity might differ from scalp EEG, particularly during locomotion when muscle activity increases.

Acknowledging uncertainty in ground truth EEG spectral content during locomotion, intrastride alpha and beta fluctuations are well reported during slow walking using EEG. Bilateral sensorimotor alpha and beta activities therefore appear to be involved in regulating stride dynamics. Although McCrimmon and colleagues [59] attributed increased primary motor cortex gamma activity to high level locomotor control processes, such as adjusting gait speed and duration, rather than sensory processing, recordings from additional brain structures are required to draw concrete conclusions. Decreased bilateral coordination has also been reported based on lower limb gait dynamics in slow compared to fast walking [62]. The authors speculated that slower gait speeds might therefore require greater attentional resources and supraspinal input [62]. Ultimately, locomotor control likely involves complex interactions among brain areas that integrate sensory and motor processes, particularly during online gait adjustments. Fortunately, high density EEG captures electrocortical signals across the scalp, which has uncovered interactive processes among cortical structures in upper limb tasks [63], [64]. Here we show that novel hardware and signal processing can enable similar advances in the study of locomotor control using mobile EEG, though additional work is needed to decode the information from these brain signals during gait. Along these lines, reduced EEG beta power was recently reported over the contralateral sensorimotor cortex during a seated knee extension task in spinal cord injured subjects [65]. This spectral power decrease during movement was followed by a spectral power increase after movement termination that further increased with spinal cord stimulation. The authors attributed this EEG spectral power change to cortical excitability modulation through proprioceptive pathways [65].

To study a wide range of locomotion speeds, we chose relatively aggressive cleaning parameters in each preprocessing method. Although these parameters are adjustable, some evidence of over cleaning was apparent using ASR, which showed reduced delta and theta spectral power in 2.0 m/s walking (Figs. 3 & 4, rows 2 & 5, right column). Low frequency spectral power reductions, however, were also observed when applying higher standard deviation cutoffs (e.g. 10 and 20). Notably, similar spectral power fluctuation patterns were apparent after applying ASR to single and dual layer EEG, compared to dual layer EEG processing using PCA and CCA. In this case, however, single layer EEG also benefitted from a passive mechanical effect of cable bundling and the overlaid secondary cap [43], [44] because all data were collected with dual layer EEG hardware. An important distinction between ASR and our dual layer EEG approach using PCA and CCA is that our artifact component selection and cleaning criteria are based on simultaneous noise recordings, rather than statistical features from clean EEG while subjects were motionless. Dual layer EEG processing can therefore be carried out without calibration or baseline comparisons, which can enable more straightforward online EEG artifact removal without assumed similarities between EEG recorded at rest and EEG recorded during motor tasks. Although each approach carries assumptions, we believe objective artifact measures can improve EEG cleaning reliability and validity. Our ability to directly compare artifact recordings to pre and post-processed EEG removes uncertainty when interpreting brain activity during movement. Artifact identification is therefore simplified during channel-level preprocessing and independent component rejection, similar to previous approaches that identified and filtered components based on accelerometer data [66], [67]. Our dual layer EEG approach, however, is well-suited for removing electrical artifacts [42]. Dual layer EEG processing might also benefit from alternative cleaning methods, including adaptive filtering [68]. Future signal processing evaluations should nevertheless include benchmark tests with ground truth signals broadcast through electrical head phantom devices during motion [43], [44], [69], [70].

In the current EEG processing approach, we applied frequency domain noise cancellation to artifact related principal and canonical components. Frequency domain noise cancellation was also applied directly to EEG channel data, but was less effective at removing artifacts that masked gait related electrocortical fluctuations (Figs. 3 & 4, row 4). Component decomposition methods prior to EEG cleaning therefore appeared to be more effective at isolating noise. We elected to clean rather than reject artifact related components in order to limit over cleaning and data rank reductions prior to ICA. Related to these concerns, we acknowledge contention surrounding PCA dimension reduction prior to ICA [71], but do not believe this step dramatically altered our ICA-derived brain sources during dual layer EEG processing. We do, however, note fewer subjects and components contributed to our cortical clusters after dual layer processing (Figs. 3 & 4, left column), which is likely due to additional artifact component rejection steps. Ultimately, these steps helped to ensure our electrocortical clusters were free of artifacts.

## V. Conclusion

Human sensorimotor electrocortical dynamics changed with gait speed, revealing lateralized sensorimotor activity tied to gait events. Intrastride electrocortical activity showed left and right sensorimotor alpha and beta power increased in contralateral limb single support and push off, and decreased during swing at each gait speed. At faster speeds, spectral power fluctuations had limited duration and bandwidth, along with reduced alpha and beta power across the gait cycle, after dual layer EEG motion and muscle artifact removal. Reduced sensorimotor spectral power could be indicative of greater cortical resources attuned to sensory feedback at faster locomotion speeds. This would prime sensorimotor cortices for performing sudden gait adjustments. Using our dual layer EEG hardware we were able to quantify artifact sources and clean noisy data. Isolated noise recordings showed discernible spectral power fluctuations from electrocortical activity after preprocessing, which helped rule out the effects of motion and muscle artifacts. Dual layer EEG can help expand possibilities for studying human brain activity in dynamic tasks.

## Supplementary Material

supplementaryfigures
