# Supplementary material for: Faster gait speeds reduce alpha and beta EEG spectral power from human sensorimotor cortex
Source: IEEE Trans Biomed Eng. Author manuscript; Available in PMC 2021 Mar 1. (PMC7134343; doi:10.1109/TBME.2019.2921766)
Supplement: supplementaryfigures [file NIHMS1565487-supplement-supplementaryfigures.pdf]

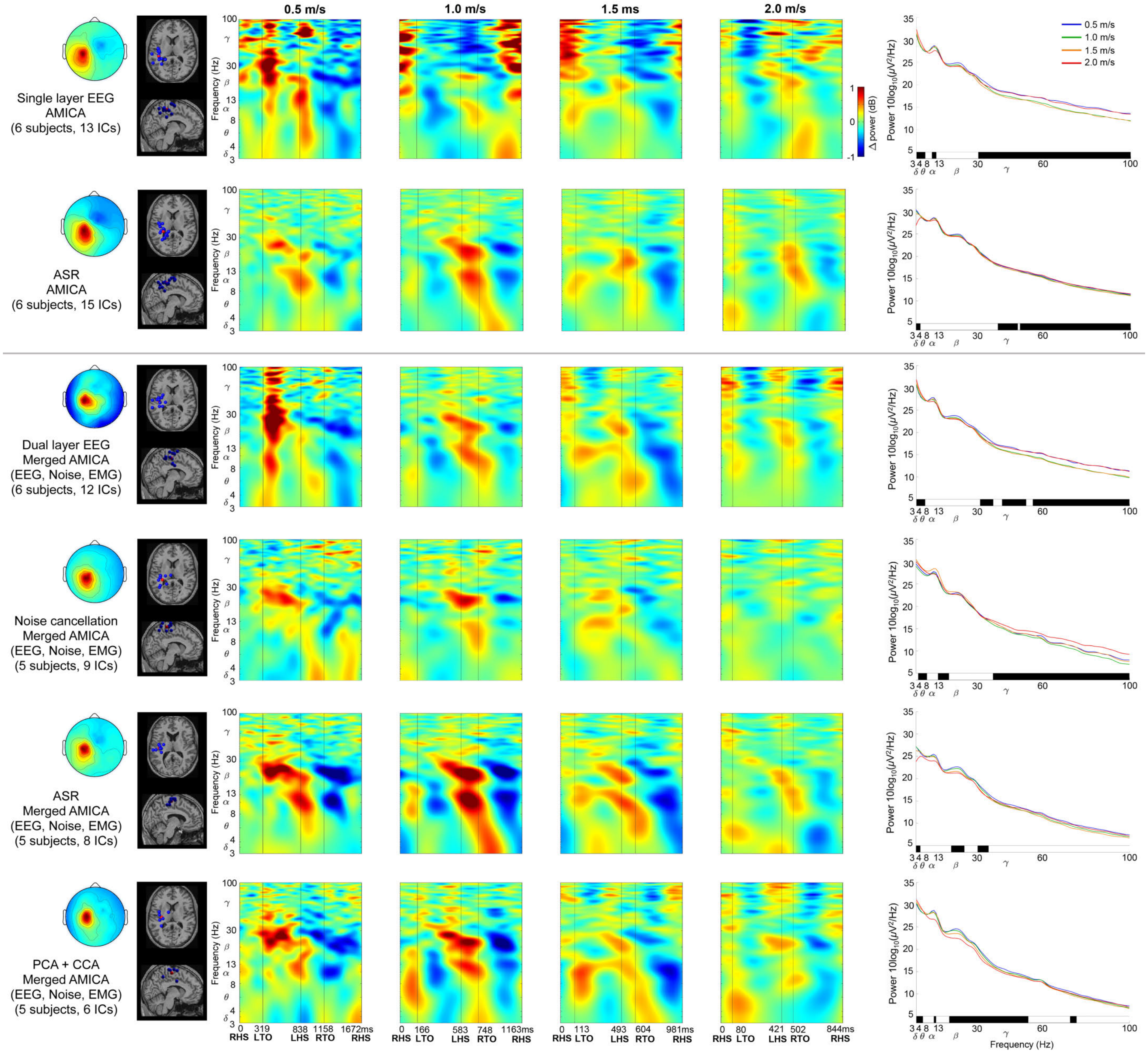

Supplementary Figure A. Left sensorimotor cortex data processing comparisons (separate processing in each row). Two traditional single-layer EEG approaches were applied to the scalp interfacing dual layer EEG sensors (top two rows) and four dual-layer EEG approaches incorporated all sensor data (bottom four rows). Left to Right: Mean cluster topographic map, Dipole locations (Blue: subject dipoles, Red: cluster centroid), Event Related Spectral Perturbation plots at each walking speed (R: right, L: left, HS: heel strike, TO: toe off), Power spectral density at each walking speed (significant speed differences at each frequency identified below each plot in black, $p < 0.05$). Equivalent ERSP plots from Fig. 3 without statistical significance masking.

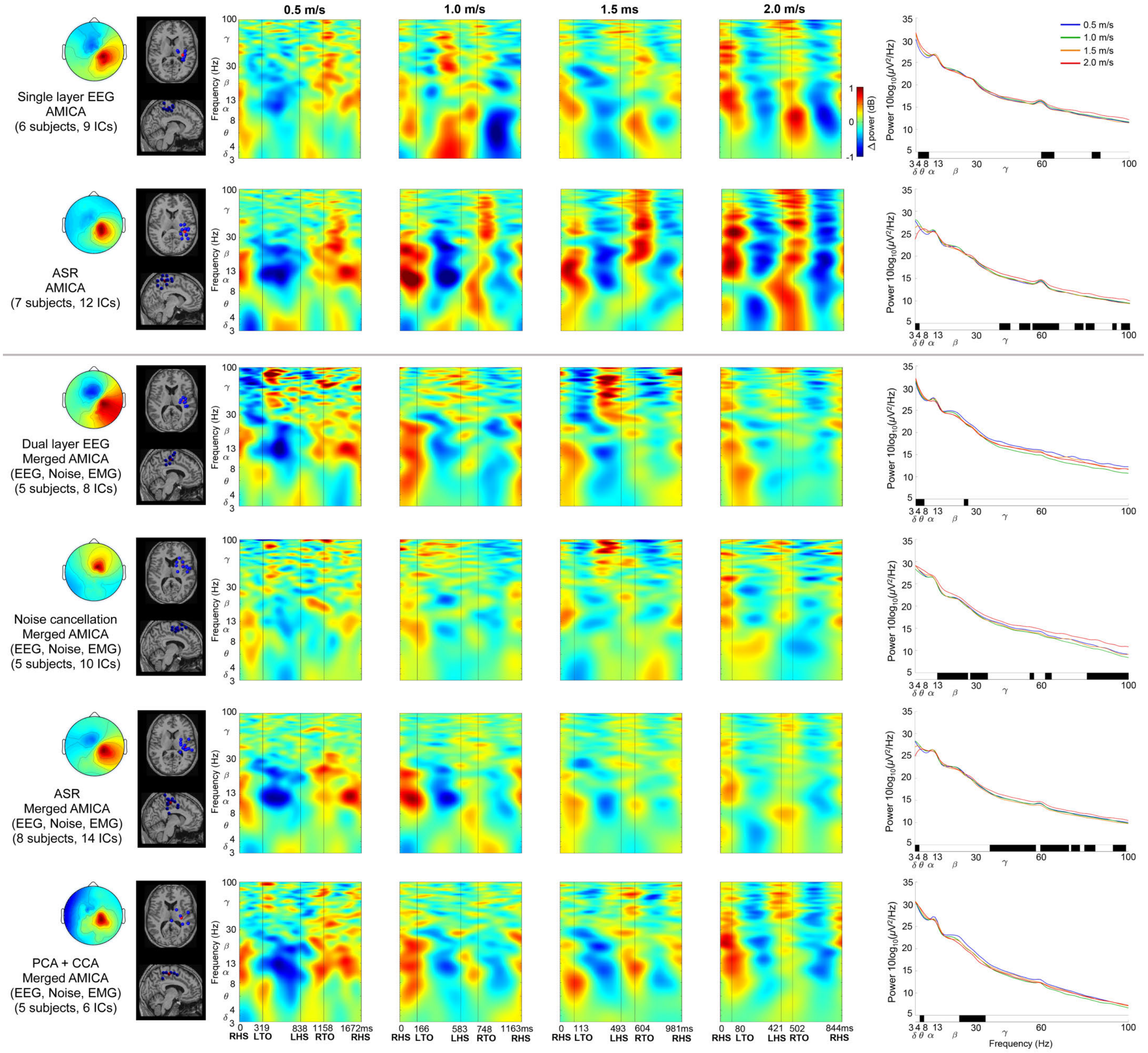

Supplementary Figure B. Right sensorimotor cortex data processing comparisons (separate processing in each row). Two traditional single-layer EEG approaches were applied to the scalp interfacing dual layer EEG sensors (top two rows) and four dual-layer EEG approaches incorporated all sensor data (bottom four rows). Left to Right: Mean cluster topographic map, Dipole locations (Blue: subject dipoles, Red: cluster centroid), Event Related Spectral Perturbation plots at each walking speed (R: right, L: left, HS: heel strike, TO: toe off), Power spectral density at each walking speed (significant speed differences at each frequency identified below each plot in black, $p < 0.05$). Equivalent ERSP plots from Fig. 4 without statistical significance masking.

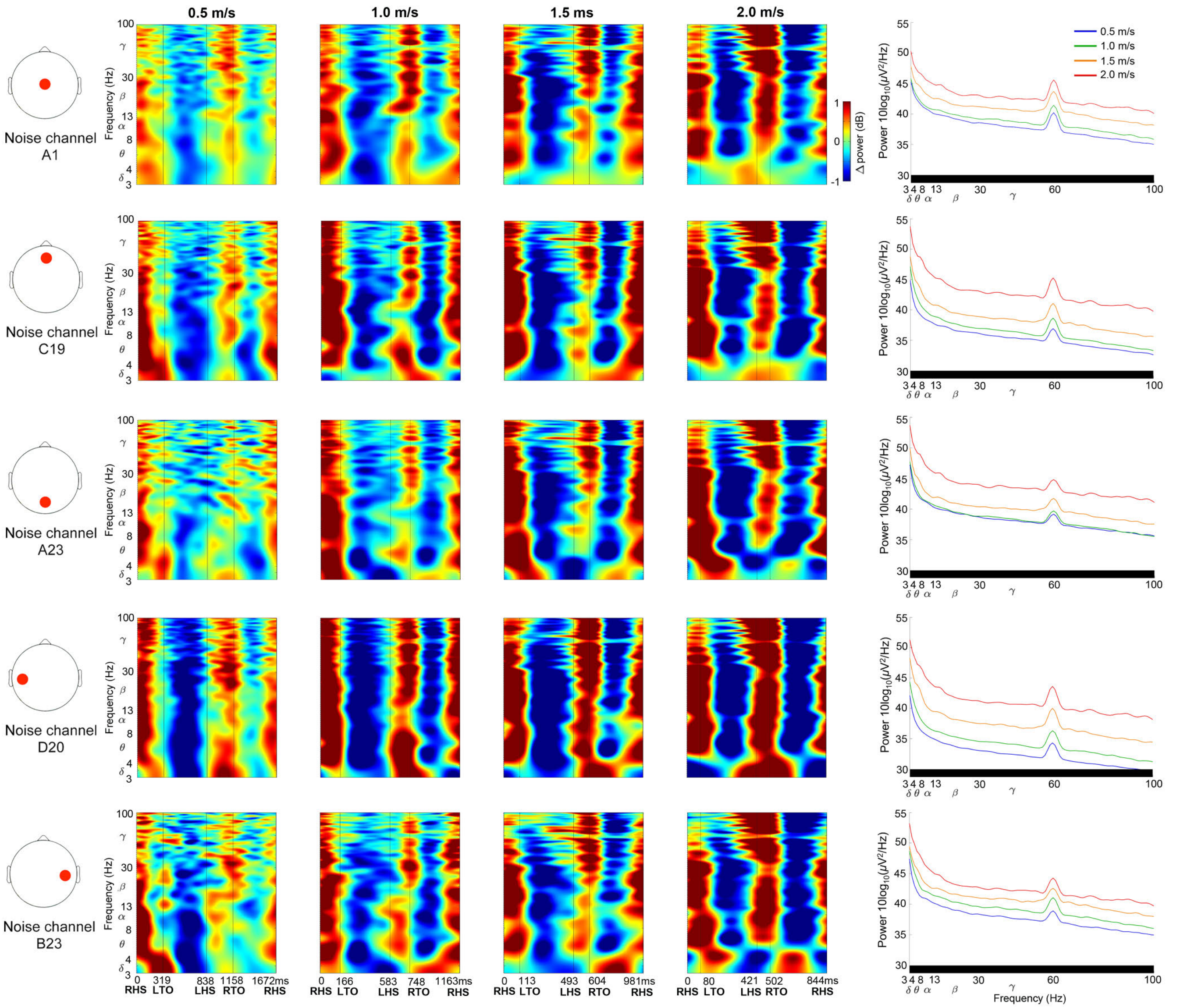

Supplementary Figure C. Exemplar dual electrode isolated noise channel data. Left to Right: Noise channel scalp location (red circle), Event Related Spectral Perturbation plots at each walking speed (R: right, L: left, HS: heel strike, TO: toe off), Power spectral density at each walking speed (significant speed differences at each frequency identified below each plot in black, $p < 0.05$). Equivalent ERSP plots from Fig. 5 without statistical significance masking.

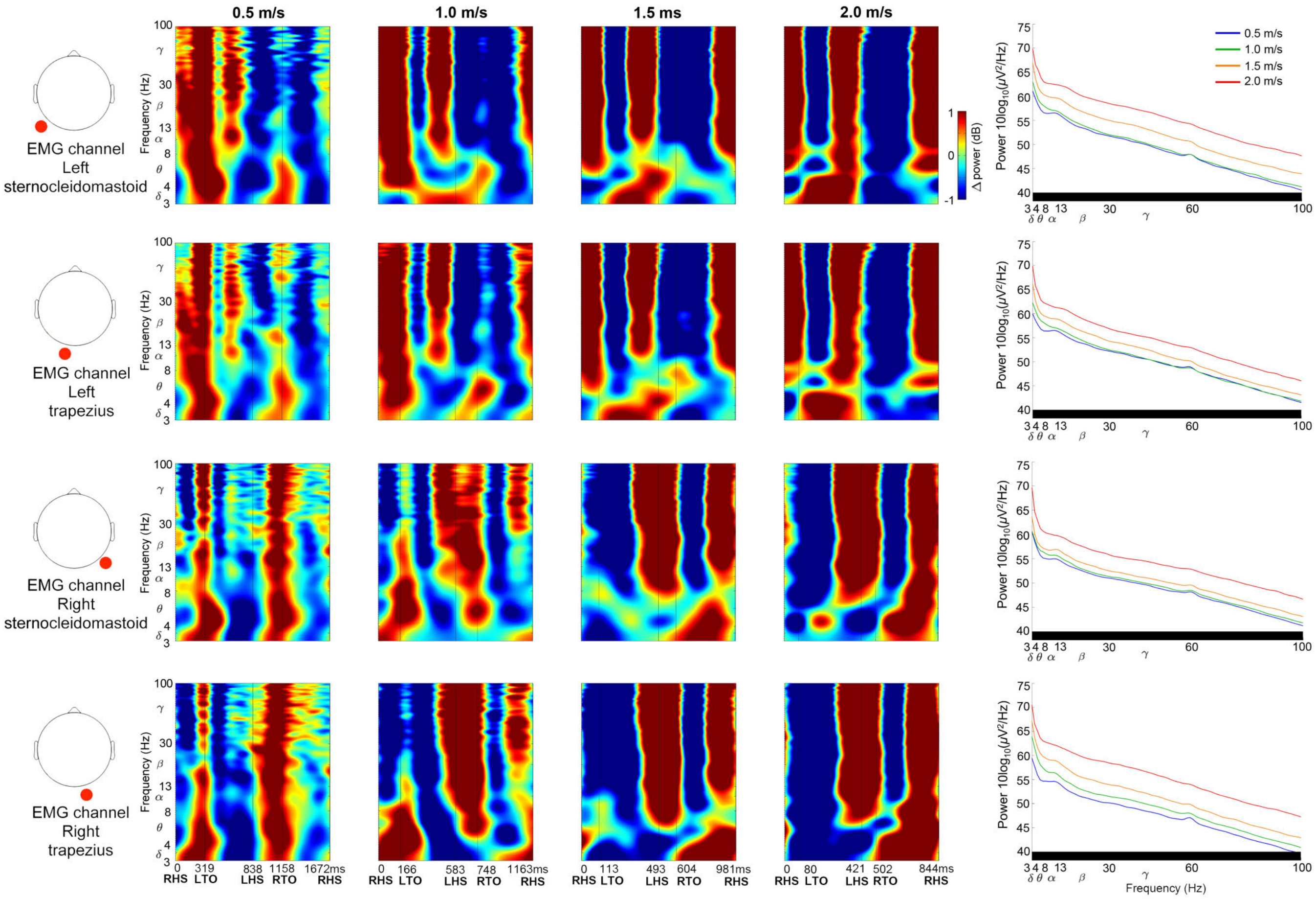

Supplementary Figure D. Exemplar neck EMG channel data (left and right sternocleidomastoid and trapezius muscles). Left to Right: EMG channel location (red circle), Event Related Spectral Perturbation plots at each walking speed (R: right, L: left, HS: heel strike, TO: toe off), Power spectral density at each walking speed (significant speed differences at each frequency identified below each plot in black, $p < 0.05$). Equivalent ERSP plots from Fig. 6 without statistical significance masking.
